# Emotion-linked shopping in digital media environments: a regulatory process framework with implications for adolescents and emerging adults

**DOI:** 10.3389/fpsyg.2026.1961945

**Published:** 2026-10-28

**Authors:** Siting Jiang, Houyu Zhao

**Affiliations:** 1 Department of Student Affairs, Zhejiang Fashion Institute of Technology, Ningbo, China; 2 Department of Diving Medicine, Navy Special Medical Center, Naval Medical University, Shanghai, China

**Keywords:** adolescents, compulsive buying-shopping disorder, digital emotion regulation, emerging adults, impulse buying, media psychology, online shopping, social commerce

## Abstract

Shopping is now embedded in social media feeds, influencer content, livestreams, entertainment, and everyday smartphone use. These hybrid media-commercial environments make browsing and purchasing readily available when distress, boredom, identity concerns, or social needs arise. Research on this topic is divided across retail therapy, compensatory consumption, impulse buying, digital emotion regulation, and compulsive buying-shopping disorder, and the presence of emotion is often treated as evidence that regulation has occurred. This critical narrative review integrates these literatures while separating emotional antecedents, regulatory intentions, changes in emotion, behavioral control, and longer-term consequences. Literature was identified through seven search sources supplemented by backward and forward citation chaining. The clearest experimental evidence concerns temporary restoration of control through choice, whereas developmental evidence is dominated by cross-sectional studies. Evidence that low-friction payment or recommender systems alter emotion-shopping sequences comes mainly from adult consumer research and general digital-commerce research and is therefore treated as indirect for adolescents and emerging adults. We develop a media-amplified regulatory-process framework in which regulatory demand is followed by shopping-affordance appraisal, enactment across multiple shopping stages, immediate and delayed outcomes, and learning that shapes later strategy selection. Regulatory narrowing is proposed as a domain-specific extension of regulatory-flexibility theory: shopping occupies an increasing share of a person’s coping repertoire as alternatives become less accessible and platform feedback makes consumption easier to repeat. The framework also specifies intensive longitudinal approaches for testing within-person change and visually distinguishes the hypothesized recursive recommender-feedback pathway from the organizing process sequence. The review identifies major gaps in temporal inference, developmental sampling, measurement alignment, cultural generalizability, and platform-sensitive research.

## 1 Introduction

Shopping no longer begins only when a consumer enters a store or opens a retail website. Products now appear inside social feeds, short-form videos, livestreams, entertainment, and peer communication. A user can move from comparison or boredom to product discovery and payment without a clear boundary between social participation and consumption. This convergence makes shopping relevant to emotional life as well as material acquisition: browsing may distract, a purchase may function as a reward, and a product may be used to express identity or affiliation.

Adolescence and emerging adulthood provide an important, but often overstated, developmental context. Across these periods, autonomy expands while identity, peer relationships, emotion-regulation skills, and financial responsibility continue to change. Some adolescents make private consumer choices while remaining dependent on family resources; many emerging adults gain access to digital wallets or credit before income is stable. Products may also carry unusual symbolic weight when they signal maturity, competence, aspiration, or group membership (Arnett, 2000; Crone and Dahl, 2012; Dahl et al., 2018). These conditions make shopping psychologically meaningful, but they do not establish that young people are uniformly vulnerable or that emotional shopping is necessarily harmful.

The empirical literature offers several partial explanations. Work on retail therapy suggests that people sometimes shop with the aim of improving mood, and experiments indicate that making choices can reduce residual sadness by restoring a sense of control (Atalay and Meloy, 2011; Rick et al., 2014). Research on compensatory consumption shows how products may be recruited after threats to identity, status, or belonging (Mandel et al., 2017). By contrast, impulse-buying studies focus on rapid enactment, whereas research on compulsive buying-shopping disorder (CBSD) concerns persistent loss of control, distress, and impairment (Iyer et al., 2020; Müller et al., 2021). These traditions overlap, but they do not describe interchangeable phenomena.

Conflating them creates both clinical and conceptual errors. An affordable, planned purchase used to mark an achievement should not be classified alongside recurrent buying that produces debt, concealment, or functional conflict. At the same time, describing repeated loss of control as benign self-care understates harm. A useful account must preserve ordinary pleasure and identity expression while identifying the conditions under which consumption becomes difficult to replace or interrupt.

A second problem is temporal inference. Cross-sectional associations between distress and impulsive or compulsive buying cannot show whether distress preceded a particular episode, whether shopping altered the target emotion, or whether later regret and financial strain intensified distress. The sequence may also be shaped by impulsivity, materialism, problematic digital use, and available resources. Contemporary emotion-regulation models treat regulation as a sequence that includes detecting a need, selecting and implementing a strategy, and monitoring its effects (Gross, 1998, 2015; Sheppes et al., 2014). Each step requires evidence; a correlation between mood and buying supplies only part of that sequence.

The media-psychological contribution is not simply that shopping occurs online. Hybrid platforms can evoke an emotional state, display a purchasable response, facilitate immediate action, and learn from the resulting engagement within one interface. Digital-emotion-regulation research shows that people use smartphones and social media for distraction, connection, arousal change, and meaning-making (Smith et al., 2022; Shi et al., 2023). Adolescent studies likewise suggest that immediate effects depend on the activity, the person, and the situation (Hollenstein and Faulkner, 2024; Scott et al., 2024). Shopping deserves separate attention because the media action can end in a financial transaction whose costs appear later.

This review asks three questions. First, what evidence is needed before shopping can be described as emotion regulation? Second, how do developmental conditions and platform affordances alter the likelihood that shopping is selected and repeated? Third, what separates bounded emotional use from problematic reliance? The article integrates consumer psychology, affective science, developmental research, media psychology, and behavioral-addiction research. It also develops a five-stage framework and a set of testable propositions, while treating direct evidence involving adolescents as limited rather than assuming that adult mechanisms transfer unchanged.

The central position is deliberately conditional. Shopping can provide short-lived relief, reward, agency, or social meaning, yet the same episode may introduce delayed costs or leave the original problem untouched. Its significance depends on the regulatory goal, the shopping stage that carries the effect, the duration of emotional change, the consumer’s ability to stop or choose alternatives, and the accumulation of financial or interpersonal conflict.

This approach follows broader media-effects research, which has moved away from universal claims based on exposure time. Associations between digital media and adolescent well-being are generally heterogeneous, with mechanisms and differential susceptibility more informative than raw duration (Kross et al., 2021; Marciano et al., 2022; Valkenburg et al., 2022; Orben et al., 2024; Sala et al., 2024). Commercial access is therefore best treated as an opportunity structure whose effects depend on proximal regulatory demands and platform features as well as broader financial, developmental, and cultural contexts.

## 2 Review approach and evidentiary standards

A critical narrative review was chosen because the relevant evidence is distributed across several disciplines and uses constructs that are similar in language but different in meaning. The review compares claims across these literatures, distinguishes direct from indirect evidence, and develops a framework for empirical testing. This approach is consistent with review-method guidance for broad, fragmented literatures and with narrative-review quality principles that emphasize transparent search description, critical referencing, and explicit presentation of evidence level (Snyder, 2019; Baethge et al., 2019).

Searches were updated through 8 September 2026. Seven search sources were used: PubMed/MEDLINE and Google Scholar, together with targeted searches of Frontiers, SpringerLink, ScienceDirect, Wiley Online Library, and Emerald Insight. Backward and forward citation searches were conducted from foundational and recent review articles. Buying-related terms (shopping, retail therapy, compensatory consumption, impulse buying, compulsive buying-shopping disorder, online shopping, social commerce, and livestream commerce) were combined with emotion-related terms (emotion regulation, mood repair, coping, distress, loneliness, boredom, anxiety, reward, identity, and control) and developmental or media terms (adolescent, emerging adult, young adult, social media, smartphone, influencer, platform, recommender system, digital payment, and deferred payment).

Practical eligibility boundaries guided study selection. Priority was given to peer-reviewed experiments, intensive longitudinal studies, systematic reviews and meta-analyses, validated measurement studies, and recent empirical work involving adolescents or emerging adults when shopping-related outcomes were measured. Adult experimental or clinical studies were retained when they tested a mechanism central to the framework, and older studies were retained when they introduced constructs that remain foundational, including emotion regulation, consumer socialization, retail therapy, compensatory consumption, impulse buying, payment salience, and regulatory flexibility. Studies were not used as core evidence when they addressed commercial optimization without a relevant psychological construct, lacked sufficient methodological information, or were redundant with a more informative report. Peer-reviewed conference work was retained only when it directly addressed a mechanism not adequately represented in the journal literature, such as recommender-system feedback dynamics (Jiang et al., 2019).

The narrative search was documented through the search sources, search terms, citation chaining, and final synthesis. Record-level counts of identified and screened references are unavailable from the original workflow. The final review cites 127 sources, and seven direct developmental studies are summarized in Table 1. Evidence was interpreted at three levels: direct developmental evidence from adolescent or emerging-adult samples with shopping-related outcomes; mechanism evidence from adult experiments, clinical studies, or digital-emotion-regulation research; and contextual evidence concerning media affordances, financial socialization, payment design, culture, and developmental sensitivity. Cross-sectional indirect effects were interpreted as statistical associations consistent with a proposed pathway; temporal mediation requires longitudinal evidence (Maxwell and Cole, 2007; O’Laughlin et al., 2018).

**Table 1:** Direct developmental evidence on emotional, digital, and self-regulatory correlates of shopping.

Study and sample	Design/context	Focal variables	Main finding	What the study can and cannot establish
Etxaburu et al. (2024); 1,498 Ecuadorian adolescents, 14–18 years (M = 15.77)	Cross-sectional school survey	Buying-shopping symptoms, attachment, emotional dependence, impulsivity	Preoccupied attachment showed a cross-sectional indirect association with buying-shopping risk through emotional dependence at medium or high impulsivity.	Direct adolescent evidence of correlated vulnerabilities; does not establish episode-level emotion regulation, temporal mediation, or clinical diagnosis.
Chen et al. (2022); 479 Chinese college students	Cross-sectional survey; structural equation modeling	Active/passive social media use, enjoyment, depression, impulse buying, materialism, self-control	Enjoyment and depression were associated with impulse buying; passive use was also associated with depression.	Supports covariance among media use, affect, and buying tendency; the cross-sectional indirect-effect model does not establish direction or within-person sequence.
Ling et al. (2023); 568 Chinese undergraduates (M age = 19.58)	Cross-sectional online survey	Upward social comparison, materialism, envy, online compulsive buying	Materialism and envy showed cross-sectional statistical indirect associations between upward comparison and online compulsive buying.	Identifies cognitive and affective correlates; cannot establish temporal mediation, emotional improvement, or disorder.
Nyrhinen et al. (2023); 1,000 Finnish young adults, 18–29 years	Cross-sectional population survey	Self-regulation, difficulty regulating smartphone use, online shopping addiction, indebtedness/financial dissatisfaction	Lower self-regulation and smartphone-regulation difficulties were associated with problematic online shopping and financial consequences.	Links digital self-regulation to financial harm; does not determine whether smartphone problems precede shopping problems.
Jabutay and Limpachote (2024); 394 young adults	Cross-sectional social-commerce survey; structural equation modeling	Social isolation, stress, hedonic browsing, e-impulse buying	Isolation was associated with stress and hedonic browsing; hedonic browsing was associated with e-impulse buying.	Supports a plausible coping pathway in social commerce; retrospective covariance cannot establish the sequence of isolation, browsing, relief, and purchase.
Estévez et al. (2025); 493 young adults, 18–35 years (M = 22.60)	Cross-sectional nonclinical survey	Hopelessness, alexithymia, compulsive buying	Alexithymia showed a cross-sectional statistical indirect association between hopelessness and compulsive buying.	Supports affective vulnerability as a correlate; the broad age range and cross-sectional design limit developmental and causal inference.
Sadiq and Ahmad (2026); 311 Chinese young adults, 18–35 years	Cross-sectional online social-commerce survey; structural equation modeling	Loneliness, life satisfaction, self-esteem, FoMO, online impulse buying	Higher loneliness and lower life satisfaction were linked to online impulse buying through cross-sectional indirect associations involving self-esteem and FoMO.	Provides recent young-adult evidence on social-affective correlates; sequential mediation remains temporally unverified and the broad age range limits developmental specificity.

Cross-sectional indirect effects are described as statistical indirect associations and are not interpreted as temporal mediation (Maxwell and Cole, 2007; O’Laughlin et al., 2018).

Study appraisal was qualitative because the synthesis spans heterogeneous experimental, observational, clinical, and review designs. For developmental claims and Table 1, appraisal focused on design and temporal ordering; developmental specificity of the sample; use of validated or clearly defined measures; sample coverage and age range; and alignment between the measured construct and the claim being made. The first author led the literature searches and initial conceptual categorization; the second author reviewed key full texts underpinning the developmental evidence, central mechanism claims, and the final conceptual categories. The workflow used collaborative review of key full texts and iterative conceptual refinement. Searches were expanded until additional sources no longer altered the major conceptual distinctions or the assessment of evidence gaps.

## 3 Conceptual boundaries and evidentiary claims

### 3.1 What would count as emotion regulation?

Three levels of evidence are useful. Emotion-linked shopping describes covariation between an emotional state and browsing or buying. Emotion-motivated shopping adds a reported intention or expectation to feel better, escape discomfort, reward oneself, or sustain excitement. Emotion-regulatory shopping requires evidence that a specific shopping activity changed the emotional trajectory. The strongest designs measure emotion immediately before and after an identifiable episode and specify whether the active component was browsing, choosing, paying, anticipating delivery, or using the product (Shiffman et al., 2008).

This distinction matters because several plausible sequences produce the same cross-sectional correlation. Distress may create urgency without a conscious mood-repair goal. A person may deliberately shop and experience no improvement. Another person may feel better briefly but later experience guilt or financial strain. Emotion-regulation research therefore evaluates context, timing, and feedback rather than assigning a fixed adaptive value to a strategy (Bonanno and Burton, 2013; McRae and Gross, 2020). Adolescent work reaches a similar conclusion: strategy choice and flexibility are more informative than a simple deficit model (Compas et al., 2017; Haag et al., 2024).

Accordingly, four questions should be kept separate: What emotion or self-relevant concern preceded the episode? What change did the person seek? What changed in the short term? What costs or benefits followed after the episode ended? A study that answers only the first question should not be interpreted as demonstrating successful regulation.

### 3.2 Shopping as an episode rather than a single act

Most measures reduce shopping to expenditure, purchase frequency, or a trait score. The lived episode is more complex: exposure, browsing, imagining ownership, comparing, saving an item, adding it to a cart, paying, tracking delivery, using or displaying the product, and deciding whether to keep or return it. Evidence for stage-specific functions is uneven. Adult experiments provide direct support for choice-related restoration of perceived control (Rick et al., 2014), while media-coping and digital-emotion-regulation research supports attentional displacement as a plausible function of digital activity (Wolfers and Schneider, 2021; Shi et al., 2023). Compensatory-consumption and social-exclusion studies support identity- and affiliation-related functions of product choice (Gao et al., 2009; Mead et al., 2011; Mandel et al., 2017). By contrast, propositions that product comparison creates order, checkout terminates uncertainty, or delivery anticipation extends reward are hypotheses generated by the present process account rather than established stage-specific effects. They require direct within-episode testing.

The distinction between shopping and buying is especially important online. One person may browse to manage boredom and leave without purchasing. Another may be interrupted by an influencer recommendation and buy without having sought emotional relief. A third may plan a modest self-reward after completing a task. When studies record only the final transaction, these pathways become indistinguishable. Prevention also changes depending on the active stage: spending limits address payment, whereas notification controls or feed design address exposure and initiation.

### 3.3 Adjacent constructs and why the distinctions matter

Retail therapy refers to shopping undertaken with a mood-related motive (Kang and Johnson, 2011). Compensatory consumption begins with a perceived discrepancy in identity, status, control, or belonging and uses products for symbolic repair (Gao et al., 2009; Mandel et al., 2017). Impulse buying describes a mode of enactment marked by a sudden urge and limited deliberation (Rook, 1987; Rook and Gardner, 1993). CBSD concerns persistent excessive buying, diminished control, distress, and impairment (Müller et al., 2019, 2021). The constructs can co-occur, but each answers a different question.

A mood-related purchase may be planned, affordable, and easily abandoned; an impulse purchase may arise from excitement rather than distress; and a person with CBSD may buy in response to several affective states while also showing craving, concealment, and functional impairment. Meta-analytic evidence on impulse buying further indicates that affect operates together with cognitive, dispositional, and market factors (Iyer et al., 2020). Treating all emotionally charged buying as one continuum conceals both ordinary variation and clinically important loss of control.

A useful evaluation therefore considers regulatory intention, immediate effectiveness, behavioral flexibility, and longer-term adaptiveness. These dimensions need not align. A purchase may improve mood without resolving the precipitating problem; a deliberate purchase may remain adaptive even when emotion contributed to it; and an infrequent episode can still be harmful if it involves severe financial or interpersonal conflict.

Behavioral flexibility is particularly informative. It is visible in the ability to delay, stop, browse without buying, switch to another strategy, and return to the original problem. Table 2 summarizes the boundaries among the main constructs and the claims that each measure can support. Immediate mood improvement indicates that a behavior may be reinforced; it does not by itself settle whether the pattern is beneficial over time.

**Table 2:** Conceptual boundaries among adjacent shopping constructs.

Construct	Defining question	What it does not establish	Representative sources
Emotion-linked shopping	Does emotion covary with browsing or buying?	A regulatory motive or emotional improvement	Gross (2015); Thomas et al. (2024)
Emotion-motivated shopping	Was shopping selected with an intention or expectation to change emotion?	That the target emotion actually changed	Atalay and Meloy (2011); Kang and Johnson (2011)
Emotion-regulatory shopping	Did a shopping stage alter the emotional trajectory?	Long-term adaptiveness or absence of cost	Rick et al. (2014); Müller et al. (2012)
Compensatory consumption	Does consumption address a threatened identity, control, status, or belonging need?	Generic mood repair or impulsive enactment	Gao et al. (2009); Mandel et al. (2017)
Impulse buying	Was the purchase sudden, urge driven, and weakly deliberated?	An emotional-regulation goal or clinical disorder	Rook (1987); Iyer et al. (2020)
Compulsive buying-shopping disorder	Is there persistent dyscontrol, distress, and functional impairment?	That ordinary emotional shopping inevitably progresses to disorder	Müller et al. (2019, 2021); Luigjes et al. (2019)

## 4 Developmental relevance and direct evidence

### 4.1 Identity, autonomy, and financial context

Consumer goods can support developmental exploration because they communicate who a young person is, where they belong, and who they hope to become. During adolescence, material symbols may become salient while self-definition is being negotiated and peer evaluation carries unusual weight (Chaplin and John, 2007, 2010). In emerging adulthood, purchases may mark independence, occupational identity, competence, or entry into valued social roles. These functions explain why products can matter emotionally without implying dysregulation.

The same symbolic capacity creates opportunities for compensation. When a gap between the current and desired self becomes salient, products associated with status, attractiveness, competence, or group membership can appear to narrow that gap (Mandel et al., 2017). The benefit lies partly in the meaning attached to the object, not only in ownership; work on object attachment similarly links possessions to emotion-regulatory functions across development (Timpano and Port, 2021). Deliberate identity exploration can be developmentally ordinary; concern increases when repeated acquisition becomes a principal source of self-worth or when the promised identity change repeatedly fails to materialize.

Social exclusion and comparison offer a related route. Experimental work shows that exclusion can increase spending on products believed to facilitate affiliation (Mead et al., 2011). In digital settings, influencer comparison and parasocial familiarity can make products appear socially diagnostic (Mundel et al., 2024). Direct evidence that a purchase subsequently restores belonging is much thinner. Studies should therefore ask which identity or relationship is at stake, what outcome is expected from the product, and whether the anticipated social change occurs.

Developmental interpretation requires restraint in both directions. Style experimentation, self-presentation, and celebration should not be pathologized. Conversely, cultural normality does not make a pattern harmless when it produces repeated conflict or displaces other sources of competence and connection. Materialistic values are associated on average with lower well-being (Dittmar et al., 2014; Kasser, 2016), but population-level associations cannot classify a particular purchase.

Shopping also offers a bounded experience of agency. Searching, comparing, and completing a transaction can feel manageable when educational, family, or relational circumstances do not. Consumer autonomy expands gradually (John, 1999), and access to a private smartphone or payment credential may arrive before stable income and responsibility for delayed costs. This partial autonomy complicates the meaning of benefit and harm. A purchase may symbolize independence although the money comes from parents, scholarships, or unstable work. Relief or control can coexist with later guilt, reduced resources, or family conflict. Evidence that payment mechanisms alter spending salience remains relevant (Soman, 2001; Prelec and Simester, 2001), but contemporary digital wallets and deferred-payment systems require direct developmental study rather than unqualified extrapolation.

Family financial socialization shapes how money, mistakes, and self-control are understood. Parental modeling, explicit instruction, and supervised practice are associated with later financial knowledge and behavior (Gudmunson and Danes, 2011; LeBaron and Kelley, 2021). Financial education improves knowledge and can improve behavior, although effects vary with timing and context (Fernandes et al., 2014; Kaiser et al., 2022). Programs focused on emotional shopping should connect financial decisions to the social and affective situations in which knowledge must be used.

The form of impairment also changes with development. In a financially dependent adolescent it may appear as concealment, use of family funds, repeated conflict, or loss of trust. In an emerging adult it may include missed bills, accumulated deferred payments, or interference with education and work. Purchase count and expenditure are meaningful only in relation to resources, legal access, household expectations, and competing obligations.

### 4.2 Regulatory development and heterogeneity

Emotion-regulation capacities continue to develop across adolescence, but the trajectory is not a simple movement from poor to good regulation. Young people acquire more complex strategies while their use remains sensitive to arousal, social context, prior learning, and environmental opportunity (Fombouchet et al., 2023; van Lissa et al., 2022). Heightened sensitivity to social and motivational cues can support learning and exploration while also increasing the appeal of immediately available rewards (Crone and Dahl, 2012).

Shopping may be comparatively easy to initiate under emotional load because digitally mediated browsing is rapidly executable, private, and low in verbal demand. This is an affordance-based inference rather than evidence that shopping is intrinsically superior to other regulation strategies. Emotion-regulation choice depends partly on situational demands and strategy availability, while digital-emotion-regulation studies show that smartphones are often selected for rapid distraction, connection, or affective change (Sheppes et al., 2014; Smith et al., 2022; Shi et al., 2023; Ronzhyn et al., 2023). The relevant empirical question is therefore whether shopping is selected because it fits the momentary goal and whether it outperforms—or merely displaces—available alternatives.

Regulatory flexibility provides a better benchmark than reliance on any single strategy. Adult models emphasize sensitivity to context, access to a varied repertoire, and responsiveness to feedback (Bonanno and Burton, 2013). The adolescent literature is smaller and conceptually inconsistent, but it likewise identifies repertoire and context-sensitive adjustment as central components (Haag et al., 2024). Emotion-linked shopping is less concerning when a young person can delay, stop, or choose alternatives and can revise the strategy when it fails.

The immediate and delayed parts of an episode may point in different directions. Digital activity can provide temporary attentional displacement, while consumer research documents post-purchase satisfaction, regret, guilt, and financial concern as separable outcomes (Wolfers and Schneider, 2021; Beikverdi et al., 2024). A deliberate purchase may also retain practical or identity value when emotion contributed to the decision (Mandel et al., 2017). These literatures do not establish that browsing itself resolves rumination in young consumers; instead, they support measuring mood change, control during the episode, and later adaptiveness as distinct outcomes.

Neither adolescence nor emerging adulthood is a uniform category. Age is intertwined with education, income, work, living arrangements, culture, family resources, and access to credit. The idea of emerging adulthood as an extended period of exploration is itself socially and economically contingent (Arnett, 2000). University samples should not be treated as representative of young people who enter full-time work earlier, support relatives, or use different digital and financial systems. Culture warrants separate treatment because it can shape both the meaning of an emotional goal and the social meaning of consumption. Cross-cultural emotion-regulation research shows that regulation preferences and their associations with adjustment vary across cultural settings (Matsumoto et al., 2008; Ford and Mauss, 2015), while consumer research links cultural orientations to status seeking and prosocial spending (Shavitt and Cho, 2016). In adolescent samples, shopping motives and peer-related shopping patterns also differ across national contexts (Gentina et al., 2014). These findings justify retaining culture as a contextual moderator in Figure 1, but they do not establish a culture-specific emotion-shopping pathway; such pathways require direct cross-cultural, within-person tests.

**Figure 1:**
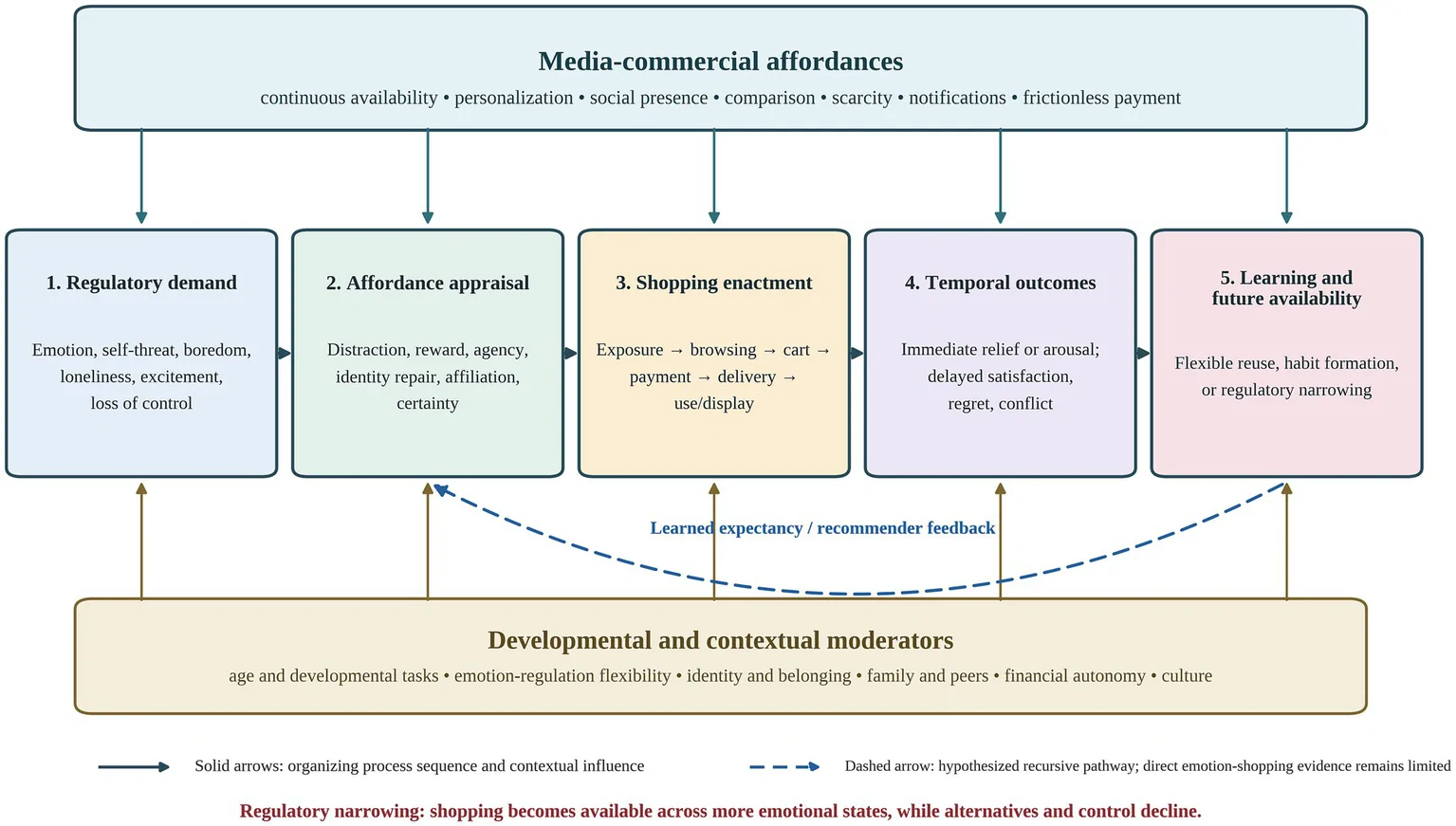
Media-amplified regulatory-process framework for emotion-linked shopping. Solid arrows indicate the organizing process sequence and contextual influence. The dashed return arrow from Stage 5 to Stage 2 denotes the hypothesized recursive pathway through learned expectancy and recommender feedback; direct evidence for emotion-specific recommender reinforcement remains limited. The figure is an organizing, hypothesis-generating framework rather than a validated causal model.

Media sensitivity is equally heterogeneous. Longitudinal and momentary studies show that relations between social media use and well-being vary across people, developmental windows, and occasions (Schemer et al., 2021; Orben et al., 2022; Steinsbekk et al., 2023; Colasante et al., 2024; Scott et al., 2024). Commercial cues are unlikely to have a uniform meaning: the same recommendation can be irrelevant to one user, socially threatening to another, and a welcome source of distraction to a third.

Digital environments also provide connection, information, entertainment, and legitimate forms of emotional support (Hamilton et al., 2023; McAlister et al., 2024). The developmental question is therefore specific: under what circumstances do commercial affordances fit a regulatory demand, and does the resulting action preserve or reduce later choice?

### 4.3 What direct developmental studies show

Direct shopping evidence remains modest and is dominated by cross-sectional surveys. The most defensible conclusion is that emotional vulnerability, social comparison, self-regulation difficulties, problematic smartphone use, and related social-affective factors covary with impulsive or compulsive shopping among adolescents and young adults. Recent evidence includes a Chinese young-adult study linking loneliness and lower life satisfaction to online impulse buying through cross-sectional indirect associations involving self-esteem and fear of missing out (Sadiq and Ahmad, 2026). The evidence is still insufficient to show that shopping reliably changes emotion within episodes or that these associations form a developmental pathway. Table 1 compares the principal direct studies and makes these limits explicit; cross-sectional indirect effects are not interpreted as temporal mediation (Maxwell and Cole, 2007; O’Laughlin et al., 2018).

## 5 Digital media affordances and strategy selection

Digital platforms do more than reproduce a store on a screen. They change the temporal, social, and attentional conditions under which shopping becomes available. In affordance terms, action possibilities arise from the relation among interface features, user goals, skills, and context (Ronzhyn et al., 2023). Infinite feeds can distract, recommendation systems reduce search effort, social signals confer validation, and checkout ends uncertainty. The same features can also intensify comparison, urgency, and repeated exposure to personally relevant cues.

Digital-emotion-regulation research shifts attention from whether media are globally helpful or harmful to the actions people choose and the effects that follow. Interviews and diary studies identify situation selection, attentional deployment, reappraisal, social sharing, and response modulation across digital activities (Smith et al., 2022; Choi and Toma, 2022). Shopping-related use can occur before a product is considered, during browsing and choice, after payment through delivery anticipation, or after acquisition through use and social display.

### 5.1 Access, personalization, and social-commercial convergence

Continuous access can compress the interval between an emotional event and a commercial action. A person who feels bored, rejected, or anxious can reach a product feed within seconds, often through the same device involved in the precipitating event. Digital-emotion-regulation research shows that smartphones and online activities are frequently selected for rapid affective change, and affordance research emphasizes that perceived action possibilities depend on availability, effort, and context (Smith et al., 2022; Shi et al., 2023; Ronzhyn et al., 2023). These findings support accessibility as a mechanism of opportunity; they do not show that shopping is the most effective response.

Personalization increases cue relevance. Recommendation systems use searches, clicks, viewing time, saved items, and previous purchases to shape later exposure. Recommender-system research demonstrates generic feedback loops in which recommendations influence user behavior and the returned behavioral data subsequently influence future recommendations (Jiang et al., 2019). Recent digital-commerce evidence also links perceived algorithmic quality and governance to impulse-buying intentions, although such work remains cross-sectional (Qiu et al., 2026). What is not yet demonstrated is the emotion-specific recursive claim that distress-linked shopping trains a recommender to present more effective commercial cues during later distress. Figure 1 therefore depicts the Stage 5-to-Stage 2 return path as a dashed, hypothesis-generating pathway rather than an equally established component of the process.

Social commerce blends interpersonal and commercial signals. Likes, comments, livestream chat, peer endorsement, and parasocial familiarity can make purchasing feel embedded in affiliation. Cross-sectional studies of college students link active and passive social media use to enjoyment, depressed affect, and impulse buying (Chen et al., 2022), while upward comparison and envy are associated with online compulsive buying (Ling et al., 2023). These findings identify candidate pathways; they cannot establish that platform exposure first altered emotion and that buying then regulated it.

Livestream commerce adds time pressure, entertainment, social presence, demonstration, and immediate checkout. Flow, social cues, and situational stimulation have been associated with impulse buying among younger users (Wang et al., 2022; Obadă and Țugulea, 2024; Xue et al., 2024; Qiu et al., 2026). A crucial distinction is whether the platform amplifies a preexisting need or generates excitement, inadequacy, or fear of missing out that would otherwise not have occurred.

### 5.2 Friction, initiation, and platform-generated affect

Stored cards, digital wallets, one-click checkout, and deferred payment can separate the moment of acquisition from the felt cost of action. Classic studies found that less salient payment mechanisms can increase willingness to spend (Soman, 2001; Prelec and Simester, 2001), and a recent meta-analysis of 392 effect sizes from 71 papers found a small but significant cashless effect on spending, with substantial contextual variation and a weakening effect over time (Schomburgk et al., 2024). This evidence is largely adult and consumer-general. It does not establish that low-friction payment specifically intensifies emotion-linked shopping among adolescents or emerging adults, so the developmental implication remains provisional.

Initiation source is rarely measured. Some episodes begin when a distressed user deliberately opens a shopping application. Others begin with a price alert, cart reminder, targeted advertisement, or influencer post. Nyrhinen et al. (2024) show that perceived relevance of targeted advertising and social-media impulsiveness are associated with online impulse buying, but retrospective surveys cannot reconstruct the exact order of cue, affect, urge, and payment. Platform logs and experience sampling are needed to distinguish user-initiated from platform-initiated episodes.

Commercial media can play at least three roles. They may amplify an existing shopping motive by reducing effort, redirect an emotional state toward products through symbolism and attention, or generate affect that subsequently promotes buying. These are different causal accounts. A cooling-off prompt may help when urgency drives action, whereas it may do little for persistent loneliness or identity threat unless alternatives are also available.

Momentary research among adolescents shows that social media can be used as distraction and that immediate effects vary by person and context (Siebers et al., 2022). Digital-emotion-regulation studies also indicate that smartphone activities are selected for rapid affective change, although selection does not guarantee success (Shi et al., 2023; Scott et al., 2024). These findings justify studying product browsing as an emotional media activity before a purchase occurs.

The broader mental-health literature offers a useful warning. Average associations between social media use and adolescent distress are small and heterogeneous, with problematic patterns and emotionally salient engagement more informative than total time (Shannon et al., 2022; Ahmed et al., 2024; Fassi et al., 2024; Sala et al., 2024). Purchase count, total spending, and platform hours are similarly blunt indicators unless they are connected to the meaning, initiation, and outcome of a particular episode.

Recent media-psychology evidence reinforces this point. Active and passive use show different and generally modest associations across well-being and social-support outcomes (Godard and Holtzman, 2024). Experience-sampling work suggests that upward comparison may be followed by lower self-esteem, whereas posting may coincide with connectedness (Desjarlais, 2024). Beliefs about whether social media are controllable tools or harmful dependencies also matter beyond exposure estimates (Lee and Hancock, 2024). Shopping research should measure perceived agency and regulatory meaning alongside behavior.

## 6 Psychological mechanisms and temporal outcomes

### 6.1 Attention and reward

The most immediate mechanism is attentional displacement. Product feeds, visual novelty, and comparison tasks can compete with rumination, worry, or boredom. Temporary distraction may be useful if it reduces escalation and the person later re-engages with the problem. Repeated use becomes more concerning when shopping functions as experiential avoidance and the underlying state remains unaddressed. Media-coping research similarly finds that motives, content, and post-use outcomes determine whether distraction is helpful (Wolfers and Schneider, 2021).

Reward can begin well before ownership. Searching, finding a bargain, building a cart, receiving confirmation, and tracking delivery may generate anticipation. Smartphone studies show that users select digital activities for rapid positive shifts (Shi et al., 2023). In shopping, the pursuit phase may therefore be more reinforcing than product use. A person can accurately report feeling better while sadness, loneliness, or practical stress remains unchanged.

### 6.2 Control, identity, and affiliation

Restoration of control has the strongest direct experimental support among the proposed mechanisms. In adult experiments, choosing products reduced residual sadness more than comparable nonchoice conditions, consistent with the idea that choice restores agency (Rick et al., 2014). The boundary is important: the studies support a short-term effect of choosing, not a general therapeutic effect of purchasing, and they do not establish the same process in adolescents.

Products can also serve identity and affiliation goals. Compensatory-consumption research shows that self-discrepancies alter product preference and that symbolic choice can restore confidence in a threatened self-view (Gao et al., 2009; Rucker and Galinsky, 2008; Mandel et al., 2017). Social exclusion can increase spending intended to facilitate connection (Mead et al., 2011). These experiments demonstrate that consumption is responsive to self and relational concerns; they provide less evidence about duration of emotional benefit or the consequences of repeated reliance.

### 6.3 Reinforcement, strategy fit, and emotion specificity

Negative reinforcement provides a plausible route from occasional use to repetition. If browsing or buying reliably reduces an aversive state, the probability of selecting it again should increase. Clinical and diary research on compulsive buying reports affective triggers, relief, and subsequent negative consequences (Müller et al., 2012; Williams and Grisham, 2012). However, relief can be reported retrospectively after a well-learned pattern has developed, so prospective studies are required to establish how reinforcement unfolds.

Self-control is often invoked as a stable explanation, but the demand for control is produced jointly by the person and the environment. Urgency, fatigue, materialism, available money, cue frequency, and interface friction all alter the difficulty of stopping. Chronic impulse buying has been described as dysfunctional self-regulation (Fenton-O’Creevy et al., 2018), and low self-regulation together with difficulty regulating smartphone use are associated with online shopping addiction and financial dissatisfaction among Finnish young adults (Nyrhinen et al., 2023). This is important evidence of co-occurrence, although the cross-sectional design leaves direction unresolved.

Different emotions also imply different goals. Sadness may make agency attractive; anxiety may increase the appeal of closure and certainty; boredom may increase novelty seeking; loneliness may direct attention toward affiliation; and identity threat may increase preference for symbolic products. Research that combines these states into a single negative-affect score cannot determine whether a platform feature or product actually matches the regulatory demand. Emotion-specific hypotheses should be tested within episodes.

Positive affect deserves equal attention. Celebration, pride, and excitement can motivate planned self-reward, and positive urgency can also promote rapid action (Rose and Segrist, 2014). The relevant distinction is not positive versus negative emotion but whether the purchase remains deliberate, affordable, and consistent with longer-term goals. Across these mechanisms, evidence strength is uneven. Control restoration has experimental support in adults; identity and affiliation mechanisms are supported by consumer experiments; reward and distraction are plausible from digital-regulation research; and recursive algorithmic reinforcement remains largely a theoretical extension. The framework below preserves these differences rather than treating all pathways as established.

## 7 From bounded use to problematic reliance

### 7.1 When shopping can help—and what the evidence supports

*Durability matters*. A product that is used, valued, and affordable can carry benefits beyond the moment of purchase. By contrast, repeated orders that are unopened or returned may reveal that anticipation rather than ownership is driving the episode. Post-purchase research identifies satisfaction, regret, guilt, disappointment, financial concern, and relationship consequences as distinct outcomes rather than a single positive–negative dimension (Beikverdi et al., 2024).

The most informative protective feature is retained choice. A person who can postpone payment, leave a cart, reduce exposure, or use noncommercial alternatives is less constrained than someone who experiences shopping as the only credible route to relief. Frequency alone cannot capture this difference.

### 7.2 Reinforcement, automaticity, and cumulative conflict

Repeated short-term benefit can make shopping easier to retrieve under later emotional load. Habit research distinguishes repetition from automatic cue-response control (Verplanken and Sato, 2011). In commercial settings, cues are also actively refreshed through notifications, targeted advertisements, and saved-cart reminders. The behavior may become increasingly available even before purchase frequency rises markedly. Conflict often accumulates outside the shopping episode. Immediate anticipation can coexist with later guilt, debt, family tension, concealment, or reduced ability to meet other goals. Mood-repair research has long shown that people may prioritize immediate affect when distressed (Tice et al., 2001). Digital payment intensifies this temporal asymmetry because the rewarding part of the event is immediate and the cost can remain abstract.

Automaticity should therefore be measured alongside control and consequences. Useful indicators include unplanned platform entry during emotion, failed attempts to delay, repeated purchase after regret, narrowing use of other coping strategies, and increasing conflict with financial or relational goals. These markers describe the process more precisely than a single threshold for shopping frequency. This pattern is related to, but not identical with, problematic social media use. Reviews indicate that problematic patterns correlate more strongly with distress than raw exposure, and impulsivity is associated with problematic use across several measures (Lewin et al., 2023; Piccerillo and Digennaro, 2025). Shopping adds a distinctive feature: the media episode can create debt, product accumulation, and repayment obligations.

A repeated emotional motive is therefore best treated as a potential learning process. Harm becomes more likely when benefits are short-lived, costs are delayed, and the consumer’s repertoire of alternatives shrinks.

### 7.3 Risk, impairment, and compulsive buying-shopping disorder

Elevated risk is indicated by a convergence of emotional triggering, automaticity, failed attempts to limit buying, concealment, short-lived benefit, and cumulative goal conflict. Adult screening traditions and recent diagnostic proposals distinguish symptom severity from ordinary purchasing (Faber and O’Guinn, 1992; Andreassen et al., 2015). Proposed CBSD criteria emphasize persistent excessive purchasing, diminished control, distress, impairment, and exclusion of better explanations (Müller et al., 2021). Screening measures developed for adults require caution in adolescents because dependence on family resources and restricted access to credit change the meaning of spending and impairment.

The available developmental findings are associative. In Ecuadorian adolescents, attachment insecurity, emotional dependence, and impulsivity were related to buying-shopping symptoms (Etxaburu et al., 2024). Among young adults, hopelessness and alexithymia have been linked to compulsive buying (Estévez et al., 2025). Adult evidence also connects adverse experiences and emotion dysregulation with problematic buying (David et al., 2024; Richardson et al., 2024).

These studies justify closer developmental work but do not establish a progression from ordinary emotional shopping to disorder. Appetitive imagery, impulse buying, craving, and impairment should also remain distinct. Mental simulation is associated with compulsive buying through impulse buying and self-control failure, but the evidence is cross-sectional and includes a broad adult age range (Duan, 2025). A theory-driven review of behavioral addictions identifies urgency and craving as affect-laden processes while noting limited evidence outside gambling (López-Guerrero et al., 2023). Process models of addictive behavior and experimental work on buying despite negative consequences provide additional hypotheses about cue reactivity and control (Brand et al., 2019; Trotzke et al., 2020). Regulatory-process concepts should guide hypotheses, not substitute for diagnostic assessment.

Demographic stereotypes are equally unhelpful. Earlier work linked compulsive buying with younger age and materialistic values (Dittmar, 2005), but treatment-seeking adults show more similarity than difference across gender in overall burden and treatment participation (Tetzlaff et al., 2026). Developmental research should report sex and gender without assuming that problematic shopping is intrinsically female or that the same products and consequences matter across groups.

The key empirical task is to identify transitions in control, strategy use, and consequences. A linear model in which ordinary retail therapy inevitably develops into CBSD is not supported by the current evidence.

## 8 A media-amplified regulatory-process framework

### 8.1 The five-stage process

Figure 1 organizes the literature as a five-stage process. An emotional or self-relevant event creates a regulatory demand. The person then appraises whether shopping offers a relevant affordance—distraction, reward, agency, symbolic repair, affiliation, or closure. Enactment may occur through exposure, browsing, imagining ownership, choosing, paying, awaiting delivery, or using the product. Immediate and delayed outcomes include relief, excitement, satisfaction, disappointment, guilt, and financial concern. Feedback from these outcomes alters the likelihood that shopping will be selected again.

The model allows different endpoints. A bounded episode may provide pleasure or temporary relief while leaving control intact. A more costly pattern develops when shopping becomes easy to retrieve across multiple states, alternatives are less available, and consequences accumulate. Developmental conditions, financial resources, identity concerns, family context, and platform design can influence each stage rather than operating as a single background moderator.

Two features are specifically media related. First, platform affordances shape appraisal as well as action: what feels like an internal shopping motive may partly reflect what the interface makes salient and executable. Second, learning may be technologically recursive. A purchase can change the user’s expectations, while clicks and transactions can change later recommendations; generic recommender-system feedback loops are established, but direct evidence for emotion-specific shopping reinforcement remains limited (Jiang et al., 2019). For that reason, Figure 1 shows the return from Stage 5 to Stage 2 as a dashed hypothesis rather than a validated causal link.

An illustrative case shows how the five stages can be measured without treating the example as evidence. After a disappointing examination result, an emerging adult may experience sadness and loss of control (Stage 1), encounter a personalized headphone recommendation and appraise browsing or choice as distraction and agency (Stage 2), browse, add the item to a cart, and pay using a stored method (Stage 3), report immediate relief but next-day financial regret (Stage 4), and later become more likely to open the platform when distressed while similar products are recommended (Stage 5). Each component can be measured separately: the precipitating state, expected function, initiation source, shopping stage, pre/post affect, delayed cost, and subsequent cue-response pattern. The case is deliberately hypothetical; its purpose is to illustrate observability and falsifiability of the framework.

### 8.2 Regulatory narrowing and its theoretical boundaries

Regulatory narrowing is proposed here as a domain-specific extension of regulatory-flexibility theory, not as a replacement for it. Bonanno and Burton (2013) describe flexibility through sensitivity to context, access to a diverse strategy repertoire, and adjustment in response to feedback. Haag et al. (2024) show that adolescent research has focused particularly on expressive flexibility and repertoire. Regulatory narrowing identifies a more specific pattern within that broader theory: a commercially mediated behavior occupies an increasing share of the person’s regulatory repertoire, while digital systems repeatedly increase its visibility and ease of use.

The construct should be tested through change over time rather than inferred from high purchase frequency. Candidate indicators include stronger within-person coupling between particular emotional states and shopping, reduced diversity of coping responses, decreasing ability to delay or interrupt an episode, rising expectancy that shopping is necessary for relief, and growing conflict with financial or interpersonal goals. A frequent shopper may remain flexible; an infrequent shopper may show severe loss of control. For example, an intensive longitudinal design could span approximately 2–3 weeks and combine several random prompts per day with event-contingent surveys when participants enter a shopping platform. Assessments could record momentary affect, regulatory goal, perceived shopping affordances, alternative coping strategies, initiation source, progression from browsing to cart or payment, and immediate post-episode affect; a later prompt could assess regret, financial conflict, and perceived control. This approach adapts established ecological operationalizations of context sensitivity, repertoire, and feedback responsiveness (Battaglini et al., 2022; Chen et al., 2024). Regulatory narrowing would require converging longitudinal evidence—stronger within-person emotion-shopping coupling together with declining nonshopping strategy diversity and worsening delay failure or goal conflict—not a single high-frequency score. Failure to observe these within-person changes would count against the narrowing hypothesis.

### 8.3 Testable propositions

The framework yields four provisional propositions. First, shopping should be more likely when product and platform affordances match the goal implied by the emotional state. Second, short-term relief should predict later shopping most strongly when alternatives are scarce and costs are delayed. Third, changes in flexibility, control, and goal conflict should predict harm more directly than emotional motive or purchase count. Fourth, platform features should affect both the immediate probability of enactment and the later availability of shopping through recommender feedback. Each proposition can be rejected or refined; none has yet been validated as part of a complete causal model. For example, Proposition 1 can be tested with multilevel event-level data by predicting transition from exposure or browsing to purchase from within-person affordance-goal fit while adjusting for affect intensity, available money, and trait impulsivity. A null or consistently inverse within-person association across episodes would fail to support that proposition. Intensive longitudinal methods are particularly appropriate because the hypotheses concern within-person sequences rather than between-person averages (Shiffman et al., 2008; Chen et al., 2024).

## 9 Methodological limitations and a research agenda

### 9.1 Temporal and episode-level inference

The dominant evidence is cross-sectional and variable centered. Studies often measure distress, a proposed mediator, and buying tendency in one survey, then discuss the indirect association as a mechanism. Cross-sectional mediation cannot establish temporal order and may be seriously biased when the underlying process unfolds over time (Maxwell and Cole, 2007; O’Laughlin et al., 2018). This critique applies directly to studies such as Chen et al. (2022) and Ling et al. (2023): their indirect-effect models are informative about covariance among media use, affective or cognitive variables, and buying tendencies, but they cannot establish that the proposed mediator changed first and subsequently altered buying. Accordingly, Table 1 describes these findings as cross-sectional indirect associations. Even conventional cross-lagged models can mix stable between-person differences with within-person change (Hamaker et al., 2015). The central question is usually person specific: when an individual is more distressed than usual, does shopping become more likely, and what happens to emotion afterward?

Retrospective reports add another problem. Retail therapy is a familiar cultural narrative, so participants may reconstruct motives from memorable purchases or later outcomes. Ecological momentary assessment reduces recall distance and has already been used to examine affect around buying episodes (Müller et al., 2012). Future studies should pair repeated emotion reports with consent-based traces of notification exposure, platform entry, browsing, cart addition, payment, delivery, return, and non-use.

### 9.2 Construct, sample, and exposure alignment

Measurement must match the claim. Trait impulse-buying scales assess a generalized enactment tendency; CBSD instruments assess symptoms and impairment; mood-regulation measures assess motives or expectancies. None can substitute for direct measurement of emotional change during an episode. Researchers should specify whether a variable represents antecedent emotion, regulatory goal, shopping stage, immediate effect, learned expectancy, or disorder severity.

Table 2 is intended as a safeguard against silent movement across these levels. Developmental labels need similar precision. University students are often described as adolescents, young consumers, or Gen Z despite differences in age, education, employment, income, living arrangement, parental oversight, and access to credit. Strictly adolescent, non-university, and economically diverse samples remain priorities. Studies should report who pays, what payment tools are available, and what form of impairment is possible at that developmental stage.

Media exposure should be measured at the affordance level. Screen time and self-reported platform hours are weak proxies for emotionally consequential use, and logged use often diverges from recall. Relevant variables include content type, initiation source, social context, recommendation exposure, scarcity cues, payment friction, and the sequence linking them to affect and action. Table 3 maps the principal evidence streams to the claims they can support and the designs needed next.

**Table 3:** Evidence streams, defensible inferences, and priority designs.

Evidence stream	Representative evidence	What the evidence can support	Priority next step
Digital emotion regulation	Smith et al. (2022); Shi et al. (2023); Hollenstein and Faulkner (2024); Scott et al. (2024)	Digital activities are deliberately selected for affective change; strategies vary by person and context	Link specific shopping stages to pre- and post-episode emotion
Media coping and social sharing	Wolfers and Schneider (2021); Choi and Toma (2022); Paul et al. (2023); Lee and Hancock (2024)	Media affordances and strategy–situation fit shape coping outcomes	Compare commercial and noncommercial media strategies within persons
Adolescent social media and well-being	Kross et al. (2021); Schemer et al. (2021); Valkenburg et al. (2022); Godard and Holtzman (2024); Orben et al. (2024)	Average effects are small and heterogeneous; mechanisms and differential susceptibility matter	Model shopping-specific content, motives, and individual susceptibility
Problematic social media use	Shannon et al. (2022); Fassi et al. (2024); Ahmed et al. (2024); Piccerillo and Digennaro (2025)	Problematic patterns correlate more strongly with distress than raw time	Test whether regulatory narrowing generalizes across media and shopping
Adolescent emotional responses online	Nesi et al. (2022); Siebers et al. (2022); Colasante et al. (2024); Iannattone et al. (2024); McAlister et al. (2024)	Emotional responses and coping functions are more informative than screen time	Use repeated measures around commercial exposure and purchasing
Social comparison and identity	Cai et al. (2021); Chen et al. (2022); Ling et al. (2023); Mundel et al. (2024); Sadiq and Ahmad (2026)	Comparison, self-evaluation, loneliness, and materialism are associated with impulsive or compulsive buying	Manipulate comparison or social-evaluative cues; assess identity- and belonging-specific outcomes
Social commerce and platform cues	Wang et al. (2022); Xue et al. (2024); Obadă and Țugulea (2024); Nyrhinen et al. (2024); Qiu et al. (2026)	Social presence, flow, targeted cues, algorithmic features, and boredom are associated with impulse-buying outcomes	Experimentally vary platform features and episode initiation source
Emotion and impulse buying	Iyer et al. (2020); Yu (2022); Tran (2022); Beikverdi et al. (2024)	Affect operates with cognitive, dispositional, and market factors; outcomes vary	Separate motive, enactment, and immediate versus delayed consequences
Retail therapy and compensatory consumption	Atalay and Meloy (2011); Rick et al. (2014); Gao et al. (2009); Mandel et al. (2017)	Choice, control, identity, and affiliation are plausible mechanisms	Replicate mechanisms in adolescent and digital contexts
CBSD and emotion dysregulation	Williams and Grisham (2012); Müller et al. (2012, 2021); López-Guerrero et al. (2023); Thomas et al. (2024); Duan (2025)	Affective triggers, relief, and impaired control characterize established problems	Prospectively test escalation without assuming continuity from ordinary shopping
Developmental context	Arnett (2000); Crone and Dahl (2012); Compas et al. (2017); Haag et al. (2024)	Identity, social sensitivity, autonomy, and regulatory flexibility change with development	Follow financial and platform-access transitions longitudinally
Cultural context	Matsumoto et al. (2008); Ford and Mauss (2015); Shavitt and Cho (2016); Gentina et al. (2014)	Emotion-regulation preferences and consumer motives vary across cultural settings; emotion-shopping-specific pathways remain untested	Use preregistered cross-cultural within-person designs rather than assuming national-group differences
Financial and design context	Soman (2001); Prelec and Simester (2001); Schomburgk et al. (2024); Mathur et al. (2019); Nyrhinen et al. (2023)	Payment salience and interface design alter action costs and spending; direct developmental evidence is limited	Test spending, affect, and repayment under manipulated friction in developmentally defined samples
Measurement and causal inference	Maxwell and Cole (2007); O’Laughlin et al. (2018); Hamaker et al. (2015); Fisher et al. (2018); Shiffman et al. (2008); Chen et al. (2024)	Cross-sectional and group-level models cannot establish episode-level mechanisms; EMA can operationalize within-person flexibility components	Use intensive longitudinal and person-specific models with objective traces
Prevention and self-regulation	Bonanno and Burton (2013); Thürmer et al. (2020); Vihari et al. (2022); Raugh et al. (2025)	Flexibility, implementation intentions, and mindfulness are plausible protective processes	Evaluate actual purchase behavior and long-term transfer

### 9.3 Converging designs

No single design can resolve the problem. Experiments can isolate choice, acquisition, endorsement, scarcity, and payment friction. Intensive longitudinal methods can identify within-person trigger-outcome sequences. Transaction records can quantify spending, repayment, returns, and repeated use, while interviews can recover product meaning, family context, and reasons for stopping. Strong conclusions will require convergence across these methods and replication across developmental stages and digital ecosystems. A staged research program follows from the evidence hierarchy. First, experiments and intensive longitudinal studies should prioritize momentary regulatory demand together with manipulable platform and payment features. Second, studies should model financial access, payment timing, and resource constraints as proximal contextual moderators. Third, longer longitudinal and cross-cultural designs should test whether developmental tasks and cultural context alter those within-episode relations. This sequence makes the strongest claims testable first while leaving distal moderators open to confirmation rather than assuming their effects.

## 10 Implications for digital well-being, prevention, and platform design

Prevention should begin by helping young consumers identify the regulatory demand before a transaction occurs. Useful questions are concrete: What am I feeling? What do I expect this product or shopping activity to change? Which part of the process is attractive—browsing, choosing, paying, anticipation, or ownership? What will the purchase displace? These questions create a pause without treating all emotional purchasing as pathology.

Financial knowledge is necessary but rarely sufficient. Educational interventions improve knowledge and can improve downstream behavior, although effects are variable and may weaken when instruction is distant from the decision (Amagir et al., 2018; Fernandes et al., 2014; Kaiser et al., 2022). Emotional awareness, implementation intentions, and prompts delivered near the decision point may be more useful in combination. If-then planning has reduced peer-driven impulse buying experimentally, but real-world durability remains unknown (Thürmer et al., 2020).

Advertising literacy should extend beyond recognizing sponsorship. Meta-analytic and developmental work suggests that literacy interventions improve persuasion knowledge more consistently than distal behavior, especially when advertising is embedded in entertainment or social interaction (Jeong et al., 2012; Hudders et al., 2017). Disclosures can increase recognition without neutralizing social comparison, parasocial influence, or urgency (Boerman et al., 2017; Evans et al., 2017). Young users need to understand how commercial content can create an emotional state and make a purchase appear to be the obvious next action.

Choice architecture is another intervention point. Clear separation of commercial and social content, realistic scarcity information, visible cumulative spending, nondefault deferred payment, voluntary cooling-off periods, notification controls, and cancellation procedures that are as easy as checkout can restore opportunities for reflection. Large-scale audits show that online interfaces commonly steer behavior through urgency and obstruction (Mathur et al., 2019). Added friction will not solve loneliness or identity threat, but it can prevent those states from being converted immediately into irreversible spending.

The goal of digital well-being should not be the elimination of emotionally meaningful media use or shopping. Digital spaces can support connection, self-expression, and temporary distraction, and blanket restrictions can remove benefits along with risks (McAlister et al., 2024). A more proportionate goal is to preserve the ability to recognize motives, delay action, evaluate fit, and retain noncommercial alternatives.

Platform responsibility follows from the same analysis. Systems can reduce engineered urgency, make cumulative cost visible, allow users to control commercial notifications, and avoid default forms of deferred payment. Such changes should be tested for emotional, behavioral, and financial outcomes rather than conversion alone. Particular attention is warranted for users who repeatedly engage after distress or show difficulty interrupting use.

For clinically significant CBSD, financial education alone is unlikely to be adequate. Adult treatment reviews provide the strongest support for cognitive-behavioral approaches, although trials remain small and adolescent protocols are underdeveloped (Müller et al., 2023). Developmentally adapted assessment should include family financial arrangements, co-occurring emotional difficulties, exposure to commercial media, and the specific function that shopping serves.

Intervention studies should also avoid evaluating success only through lower purchase frequency. More informative outcomes include restored choice, greater strategy diversity, reduced conflict, improved repayment, and the ability to tolerate or address the precipitating emotion without automatic shopping.

## 11 Discussion

The current evidence does not justify a general claim that shopping regulates emotion among young people. It supports a narrower account. Adults sometimes choose shopping for mood-related reasons, product choice can temporarily restore control, and self or relational threats alter consumer preferences. Among adolescents and emerging adults, emotional difficulties, social comparison, low self-regulation, and problematic smartphone use are associated with impulsive or compulsive shopping. Most of those developmental findings are cross-sectional, so they identify risk markers rather than an episode-level regulatory mechanism.

Evidence is strongest for the short-term role of choice and for links between identity-related concerns and consumption. It is moderate for associations among social-media experiences, affect, and shopping tendencies in college or young-adult samples. It is weakest for the propositions that shopping reliably improves emotion over time, that algorithms strengthen emotion-shopping coupling, and that ordinary emotional purchasing develops along a common pathway toward CBSD. Making these differences explicit is essential because an integrative framework becomes misleading when every arrow is treated as equally established.

The media perspective changes the unit of analysis. Platform design is not merely an external influence on a preexisting consumer trait. It determines which options are visible, how socially meaningful they appear, how quickly action can be completed, and when cost is experienced. It can also initiate an episode and use prior engagement to shape later exposure. These features make emotion-linked shopping a sequence of media use, appraisal, action, and feedback rather than a purchase decision considered in isolation.

The developmental implications are conditional rather than deficit based. Identity exploration, social sensitivity, and growing autonomy can support adaptive learning while also increasing the relevance of products and feedback. Average media effects conceal substantial heterogeneity, and the same is likely to be true for commercial exposure (Orben et al., 2022, 2024). The phrase ‘With Implications for Adolescents and Emerging Adults’ in the title is therefore intentional: it identifies the developmental population for interpretation and future testing, not a claim that the complete regulatory process has already been demonstrated in those groups.

Regulatory narrowing is the framework’s main theoretical proposal, but its contribution depends on careful positioning. The idea of a diverse repertoire, context sensitivity, and feedback-based adjustment comes from regulatory-flexibility theory; the present extension specifies how one commercial behavior may progressively occupy that repertoire and how digital systems may reinforce its availability. The construct will add value only if it predicts loss of choice and cumulative conflict beyond established measures of impulsivity, habit, problematic smartphone use, and CBSD symptoms.

The practical implication is similarly restrained. Emotional shopping is not a unitary target for prevention. Bounded self-reward, identity expression, and temporary distraction can coexist with healthy functioning. Intervention is more clearly warranted when shopping becomes automatic, difficult to replace, financially disproportionate, or repeatedly inconsistent with the person’s goals.

## 12 Conclusion

Emotion can be part of shopping without making the episode impulsive, therapeutic, or pathological. Digital media matter because emotional activation, comparison, product exposure, social endorsement, and payment can occur in one compressed sequence. The most useful questions concern what change the person sought, which shopping stage carried the effect, whether control and alternatives remained available, and what costs emerged later. The proposed framework links regulatory demand, affordance appraisal, enactment, temporal outcomes, and learning, while treating developmental and platform conditions as influences across the process. Its central hypothesis is that problematic reliance is marked less by emotional motivation or frequency than by narrowing choice, impaired control, short-lived benefit, and cumulative conflict.

Testing that hypothesis requires within-person, developmentally specific, and platform-sensitive research.
